# A Path to Early Diagnosis of MCI and Dementia: Integrating myMemCheck Into the Primary Care Workflow

**DOI:** 10.1093/geroni/igaa057.839

**Published:** 2020-12-16

**Authors:** William Mansbach, Ryan Mace, Theresa Frangiosa, Virginia Biggar, Meryl Comer, Steven Simmons

**Affiliations:** 1 Mansbach Health Tools, LLC, Simpsonville, Maryland, United States; 2 UsAgainstAlzheimer’s, Royersford, Pennsylvania, United States; 3 UsAgainstAlzheimer’s, Washington, District of Columbia, United States; 4 UsAgainstAlzheimer’s, Kensington, Maryland, United States; 5 Allegiance Housecalls LLC, North Potomac, Maryland, United States

## Abstract

Barriers to the early detection of mild cognitive impairment (MCI) and dementia can delay diagnosis and treatment. myMemCheck® was developed as a rapid free cognitive self-assessment tool that can be completed in the practice setting or at home to identify older adults that would benefit from a more comprehensive cognitive evaluation for MCI and dementia. Two prospective cross-sectional studies (N = 59; N = 357) were conducted to examine the psychometric properties and clinical utility of myMemCheck®. myMemCheck® evidenced adequate reliability (test-retest, r = 0.67) and strong construct validity (η2 = 0.29, discriminating normal, MCI, dementia). Receiver operating characteristic analysis evidenced an optional myMemCheck® cut score for identifying older adults with MCI or dementia (sensitivity = 0.80, specificity = 0.67, positive predictive value = 0.91, negative predictive value = 0.43). myMemCheck® explained 25% of cognitive status beyond basic patient information. We provide specific suggestions for integrating myMemCheck® into practice to optimize workflow. Study results are further interpreted in the context of two national online surveys (healthcare professionals, N = 181; consumers, N = 1740). Healthcare professionals widely agreed on the need (94%) and importance (86%) of cognitive self-assessments. Public demand for cognitive self-assessment was confirmed by consumers who trialed myMemCheck® as part of their survey participation—86% agreed on the need for a tool like myMemCheck®. Mixed methods findings suggest that myMemCheck® could fast- track the diagnostic process, facilitate appropriate referrals for cognitive and neuropsychological evaluation, reduce assessment burden in healthcare, and prevent negative outcomes associated with undetected cognitive impairment.

